# The Effects of Disturbance on Plant–Pollinator Interactions in the Native Forests of an Oceanic Island (Terceira, Azores)

**DOI:** 10.3390/insects16010014

**Published:** 2024-12-27

**Authors:** Mário Boieiro, Mariana Ferreira, Ana Ceia-Hasse, Fabiana Esposito, Renata Santos, Gabor Pozsgai, Paulo A. V. Borges, Carla Rego

**Affiliations:** 1 CE3C-Centre for Ecology, Evolution and Environmental Changes & CHANGE-Global Change and Sustainability Institute, University of the Azores, Rua Capitão João d’Ávila, Pico da Urze, 9700-042 Angra do Heroísmo, Azores, Portugal; pozsgaig@coleoptera.hu (G.P.); paulo.av.borges@uac.pt (P.A.V.B.); 2 LIBRe–Laboratory for Integrative Biodiversity Research, Finnish Museum of Natural History, University of Helsinki, 00100 Helsinki, Finland; cjrego@ciencias.ulisboa.pt; 3 IUCN SSC Atlantic Islands Invertebrates Specialist Group, 9700-042 Angra do Heroísmo, Azores, Portugal; 4 CE3C-Centre for Ecology, Evolution and Environmental Changes/Azorean Biodiversity Group, CHANGE-Global Change and Sustainability Institute, Faculty of Sciences, University of Lisbon, 1749-016 Lisbon, Portugal; marianamf.23@gmail.com (M.F.); espfabiana@hotmail.it (F.E.);; 5 CIBIO-Research Centre in Biodiversity and Genetic Resources, InBIO Associate Laboratory, School of Agronomy, University of Lisbon, 1349-017 Lisbon, Portugal; 6 CIBIO-Research Centre in Biodiversity and Genetic Resources, InBIO Associate Laboratory, Vairão Campus, University of Porto, 4485-661 Vairão, Portugal; 7 BIOPOLIS Program in Genomics, Biodiversity and Land Planning, CIBIO, Vairão Campus, 4485-661 Vairão, Portugal; 8 IUCN SSC Species Monitoring Specialist Group, 9700-042 Angra do Heroísmo, Azores, Portugal

**Keywords:** island biodiversity, pollination, native forest, Laurisilva, ecological networks, super-generalists, Azores

## Abstract

The native biodiversity of oceanic islands is threatened by human-driven disturbance and by the growing number of species introductions which often interfere with natural ecological processes. Here, we aim to evaluate the effect of anthropogenic disturbance on plant–pollinator interactions in the native forest communities of an oceanic island (Terceira, Azores, Portugal). We found that native species predominated in preserved sites compared to disturbed ones and that the extant plant–pollinator interactions were mostly dominated by generalist species. Dipterans, particularly hoverflies, emerged as the primary flower visitors, while introduced hymenopterans (*Apis mellifera* and *Bombus terrestris*) and native beetles were locally important, respectively, in disturbed and preserved sites. Human-driven disturbance seems to be responsible for the observed differences in flower abundance and species composition between sites and to drive changes in specific network metrics (namely nestedness, interaction strength asymmetry, and specialization), particularly in one of the study areas. Our findings also suggest that native generalist species provide ecological opportunities that can facilitate the establishment of introduced species through their broad interaction networks.

## Simple Summary

This study examined how disturbances caused by humans affect the interactions between plants and pollinators in Terceira Island’s native forests. Plant and pollinator native species dominate in preserved areas, while disturbed areas see a rise in introduced species of both study groups. Pollinator visits were mostly carried out by generalist species, especially hoverflies. In disturbed areas, non-native bees were key pollinators, while in undisturbed areas, native beetles played an important role. This research revealed that human disturbances alter species composition and the interactions between plants and pollinators. Native generalist species, of both plants and insects, appear to provide ecological opportunities that can facilitate the establishment of introduced species through their broad interaction networks. This study highlights how human activities change the natural biodiversity of island habitats.

## 1. Introduction

The isolated nature of island ecosystems, their relative simplicity, and their evolution disconnected from continental biotas make them attractive models for biodiversity and ecology studies [1]. For these reasons, many studies carried out in oceanic islands have explored community assembly processes and the consequences of human-driven changes (including alien species introduction) to native biodiversity and ecological interactions [1,2,3,4]. Species introductions, particularly those with invasive potential, are a major driver of the biodiversity crisis in oceanic islands, posing serious challenges to the definition and development of nature conservation strategies or initiatives and to the management of conflicts between human activities and island biodiversity conservation [2,5,6,7]. In fact, with the exponential increase in tourism and the transportation of goods to islands from the most diverse origins [8], this problem will likely worsen if no action is taken. Also, it is important to highlight the role of human-driven disturbance in paving the way for the establishment, spread, and even ecological dominance of introduced species. Many introduced species benefit directly from the environmental changes induced by human activities in natural habitats and indirectly from reduced biotic resistance of island communities to biological invasions [9,10].

The Azores archipelago has registered a considerably high increase in the number of introduced species in most islands [11,12,13,14,15], but the consequences of such introductions on native species interactions and ecosystem functioning remain to be studied [16]. Thus, it is key to assess and monitor ecological interactions on island ecosystems, particularly in natural areas classified as biodiversity hotspots (such as the Azorean native forests) and where endemic endangered species with restricted distributions may be vulnerable to the effects of disturbance and species introductions.

Many oceanic islands are known to support species-poor communities and to lack important taxonomic and functional groups that are common in the mainland as a result of their isolation [1,17,18,19]. This is also the case of the Azores, a remote (distanced 1368 km from the mainland) and young archipelago in the Atlantic Ocean, where significant changes were inflicted to native habitats and biodiversity following human colonization [20,21]. The scarce information available on pollinators and plant–pollinator interactions in the Azores reports simplified communities with no significant compositional differences between habitats, where a few generalist insect species are the most frequent flower visitors [17,19,22]. One of those studies, targeting the reproduction of four endangered endemic plants (*Azorina vidalii*, *Euphrasia azorica*, *Myosotis azorica*, and *Solidago azorica*) in Flores Island, found that these plants are not pollinator limited, which suggests that the high proportion of generalist visitors seems to be key to ensure the stability of plant reproduction [22]. The prevalence of endemic generalist pollinators (also coined super-generalists) seems to be a widespread phenomenon in oceanic islands [17,23], resulting from the low number of successfully colonizing species that led to reduced interspecific competitive interactions and broader niches. However, the key role of these generalist pollinators has been jeopardized in several island ecosystems worldwide due to the human-facilitated introduction and spread of exotic species [24,25].

A growing number of studies on plant–pollinator interactions report that introduced species may replace native ones and alter native species abundances as well as their interactions [25,26,27]. Furthermore, they may even have serious and long-term consequences on the structure and composition of island communities and on native species’ demography and survival. However, the impact of introduced species on natives and their interactions can vary from negative to neutral and even positive, and seem to depend on species identity, the stage of the invasion process, and habitat quality [1,18]. For instance, several studies showed that alien bees compete for floral resources with native pollinators, affect negatively native plant reproduction, and alter pollination networks, while other research has highlighted their positive impacts in rescuing native plant species from extinction and increasing ecosystem resilience to environmental change [28,29,30].

This work aims to evaluate the effect of human-driven changes (species introductions and disturbance) in native forest communities on species richness and composition of plants and pollinators and on their interactions. Here, we address the following questions: (1) Do plant and pollinator species richness and composition differ between preserved and disturbed sites? (2) What are the most important plants and pollinator species/groups in the Azorean indigenous forest, and are they native or introduced? (3) Do differences in the values of specific network metrics relate to sites’ disturbance levels?

## 2. Materials and Methods

### 2.1. Study Area

The Azores is a volcanic archipelago situated in the north Atlantic Ocean (between 36°55′–39°43′ N and 25°00′–31°15′ W), with nine islands and several islets. Originally, the Azorean landscapes had different natural habitat types in pristine condition, including the emblematic native forest usually named Laurisilva, but following human colonization, these natural habitats were strongly modified [3,21,31,32]. The native forest that covered almost entirely the Azorean islands upon their discovery in the 15th century was largely destroyed and replaced by farmlands, pastures, and, more recently, forest plantations (e.g., *Cryptomeria japonica*). Nowadays, the natural forest is reduced to very few and small patches (occupying ~5% of the total area of the archipelago), usually located at higher altitudes, where the orography and the climate are unsuitable for human establishment [3,4,31,32].

Fieldwork took place on Terceira, the third largest (402 km^2^) and relatively recent island in the Central Group, with an estimated geological age of 0.4 MY [33]. Compared with other Azorean islands, the native forest still covers a relatively large surface area (23 km^2^) on Terceira and includes some of the largest and more pristine native forest fragments of the archipelago [31,34]. The five native forest fragments here—Pico Alto e Biscoito da Ferraria, Caldeira Guilherme Moniz, Pico Galhardo, Serra de Santa Bárbara, and Terra Brava—harbor a large number of Azorean endemic species, some of which are exclusive to this island. Due to their importance for biodiversity conservation, these native forest fragments were included in a network of protected areas—the Terceira Natural Park.

Field data collection was carried out at two study areas, Lomba and Pico Galhardo, both included in the Terceira Natural Park. Lomba is located in Serra de Santa Bárbara, an important area for biodiversity conservation in Azores while Pico Galhardo is a small native forest fragment located in the center of the island [35,36]. At both study areas, two sampling sites (one preserved and one disturbed) were set relatively close to each other (distanced by ~500 m) to minimize geographic distance effects on community composition. The disturbed sites had one or more plant species that are considered problematic in Azorean native forests [37], namely *Erigeron karvinskianus*, *Hydrangea macrophylla*, and *Rubus ulmifolius*. These plants contribute to the degradation of habitat quality through changes in plant community composition and structure. More information on the study sites is presented in Figure S1.

### 2.2. Flowering Plant Composition and Insect Visitation Networks

Azorean plant species flower mostly between June and August [38,39]. Field data collection was carried out in 2016, from 4 to 27 July, to encompass the flowering peak in the selected sampling sites. However, the extended rainy season during that year delayed the flowering phenology, so our findings correspond to the period preceding peak flowering. The sampling methodology to assess plant–pollinator interactions followed the protocol proposed by Carvalheiro et al. [40]. Sampling points, each consisting of a 1 m radius semi-circle, were randomly chosen in each study site to assess flowering species composition, flower abundance, and insect visitation. A total of 100 sampling points were surveyed, 50 in each study area, and 25 per site. Insect surveys took place from 10 h00 to 18 h00 to cover the main period of their activity. In each sampling point, flowers were observed for 15 min periods, and all the insects contacting the plant reproductive structures were recorded. Following Carvalheiro et al. [40], we considered an inflorescence to be a flower unit from a flying insect perspective rather than by flower anatomy. During the 15 min period, all flower–visitor interactions were recorded, and insect specimens were collected for identification or to confirm species identity in the laboratory. The insect specimens were collected with sweeping nets and stored in vials with ethanol (70%). Samples were taken to the laboratory, and in a first approach, all specimens were identified at family level. For some insect visitor groups that are usually considered important pollinators, like bees, bumblebees, ants and wasps (Hymenoptera), butterflies and moths (Lepidoptera), beetles (Coleoptera), and larger-size flies (Diptera), identification was then performed to species level using several taxonomic references [41,42,43]. The insect specimens were later deposited in the Dalberto Teixeira Pombo entomological collection (DTP) at the University of the Azores (Angra do Heroísmo, Azores, Portugal). Plant identification took place during fieldwork, but for some specimens, the identification was later confirmed in the laboratory using specific literature [38]. All plant and insect visitor species were classified according to three distributional categories (endemic, native non-endemic, and introduced) following [20,44]. The list of plants and insects identified at each study site is presented in Tables S1 and S2, respectively.

### 2.3. Data Analysis

The differences in plant species richness, flower availability, and insect visitor richness between study areas and sites were assessed by hierarchical ANOVA, with site as a nested factor in the study area. Before performing the analyses, the data were tested for normality and homoscedasticity, and some variables (flower availability and insect visitor richness) were transformed (using the logarithmic transformation) to meet the assumptions of ANOVA. The significant differences found with ANOVA were then assessed by multiple comparisons of means using the Tukey HSD post-hoc test with a 95% confidence level. These statistical analyses were performed using the SPSS software (version 29.0) [45].

The distribution of insect visits to flowers of the different plant species in the four study sites was analyzed as separate bipartite networks (data from the 25 sampling points in each site were pooled). Bipartite networks display the interactions between insect visitors and plant species using the information on the observed number of visits. We carried out preliminary analyses with all insect visitors and flowers, but then, considering that some visitors are not important as pollinators (being frequently excluded from pollination network analyses) [46], we performed the analyses on a subset including the most commonly studied pollinator groups (e.g., bees, bumblebees, beetles, butterflies, ants, wasps, and larger-size flies).

Several network metrics that provide complementary ecological information were computed to characterize site-level pollination networks and identify differences that may relate to the degree of disturbance. Nevertheless, we are aware that network structure and properties may be influenced by network size and sampling methodology [47]. The selected network metrics were: connectance, i.e., the proportion of realized interactions relative to all possible interactions, serving as an indicator of network complexity; nestedness, a measure of the hierarchical organization of networks considering niche width; specialization that allows for assessment of the degree to which species in the network interact exclusively with a subset of other species; and interaction strength asymmetry, a measurement of the imbalance in the strength of interactions [48,49]. The network metrics were calculated using the bipartite package in an R environment [50,51]. To assess if flower visitors preferred introduced plants or simply used them because they were abundant, we checked the association between insect visits and flower abundance for each plant species using Spearman correlation tests. This test was calculated by ranking both flower visitation by plant species (rank use) and flower abundance by plant species (rank availability) from each site and then assessing the association between the two ranks. This approach has the advantage of being less sensitive to incidental sampling from rare plant species. These analyses were performed using the SPSS software [45].

## 3. Results

### 3.1. Plant Species, Flower Availability, and Flower-Visiting Insects

During this study, we sampled 22 flowering plant species in the four study sites (see Table S1). Introduced flowering plant species occurred in higher numbers and were more frequent at disturbed sites, while at preserved sites, the most frequent flowering plants were native species, such as the endemics *Hypericum foliosum*, *Lysimachia azorica,* and *Tolpis azorica* (Table S1). We found small differences in average species richness of flowering plants between study areas (F = 5.44, *p* = 0.02), but not among sites in the same area (F = 0.11, *p* > 0.05) (Table 1). The average number of flowers was significantly different between study areas (F = 22.54, *p* < 0.001) with Pico Galhardo showing higher numbers than Lomba (Table 1). Furthermore, there were less flowers at the disturbed sites in both study areas than at the neighboring preserved sites (F = 19.72, *p* < 0.001).

**Figure 1:**
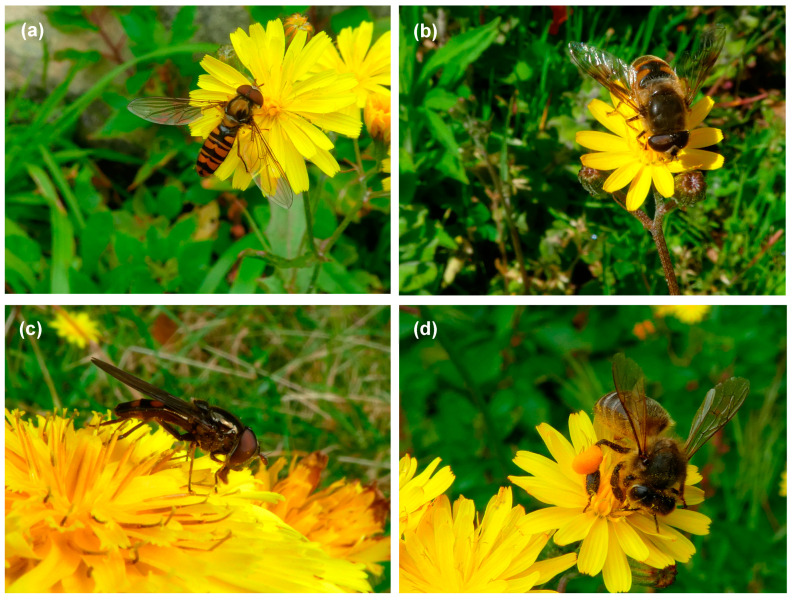
The native hoverflies *Episyrphus balteatus* (**a**), *Eristalis tenax* (**b**), and *Xanthandrus azorensis* (endemic to the Azores) (**c**) were found in all study sites, while the introduced honeybee *Apis mellifera* (**d**) was restricted to the disturbed ones.

There was a clear trend in the biogeographic origin of most flowers in the different study sites since in preserved sites, over 90% of the flowers belonged to native species (mostly Azorean endemics), while in disturbed sites, the majority of flowers (>71%) belonged to introduced species. A total of 2530 visits made by 49 flower visitor species from different insect groups were recorded at the four study sites (Table S2). Lomba had a higher overall richness of flower visitors than Pico Galhardo (Table 1), but many species were common to all study sites. For example, flower visitors such as the beetle *Anaspis proteus*, the bumblebee *Bombus terrestris*, and the hoverflies *Episyrphus balteatus*, *Eristalis tenax*, *Sphaerophoria nigra,* and *Xanthandrus azorensis* were found at all study sites (Figure 1, Table S2). Also, the average insect visitor richness was much higher at Lomba than in Pico Galhardo (F = 96.49, *p* < 0.001). Still, no significant differences were found in this variable between preserved and disturbed sites within either study area (F = 1.98, *p* > 0.05) (Table 1). The most frequent group of flower visitors at all study sites was Diptera, particularly hoverflies (Syrphidae) and blowflies (Calliphoridae), accounting for over 65% of the flower visits (Table 2). Beetles (Coleoptera) and bees (Hymenoptera: Anthophila) were also important flower visitors, and they seemed to be influenced by disturbance levels, as the former were mostly associated with preserved areas while the latter were responsible for a considerable number of visits in disturbed sites.

**Figure 2:**
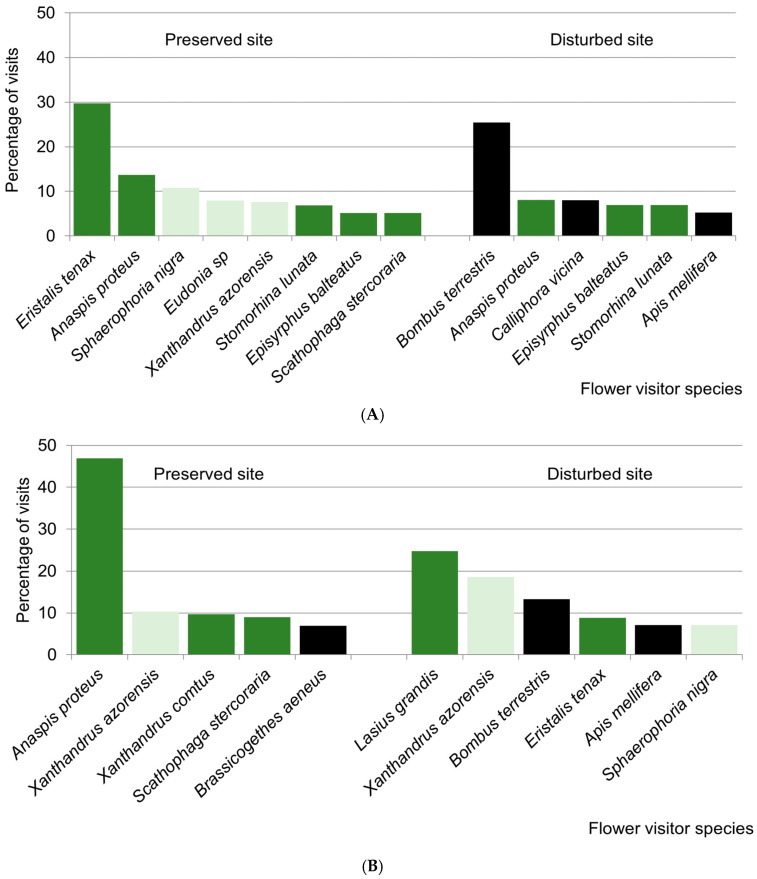
Percentage of visits made by the most important insect species (with >5% of visits) in the study sites at Lomba (**A**) and Pico Galhardo (**B**). The distribution status of each insect species (endemic in light green, native non-endemic in green, and introduced in black) is shown jointly with insect visitor species names.

Interestingly, most of the visits were carried out by native insect species, particularly in the preserved sites, and only the disturbed site at Lomba showed a relatively high percentage of visits by introduced insect species (Table 2). Native species, like the beetle *Anaspis proteus* and the hoverflies *Eristalis tenax*, *Sphaerophoria nigra,* and *Xanthandrus azorensis*, made most visits to flowers in preserved sites (Figure 2). These species were also key visitors in disturbed sites, but here, introduced species, like the bumblebee *Bombus terrestris*, the honeybee *Apis mellifera,* and the blowfly *Calliphora vicina*, played a similarly important role (Figure 2).

### 3.2. Insect Visitation Networks and Plant–Insect Associations

**Figure 3:**
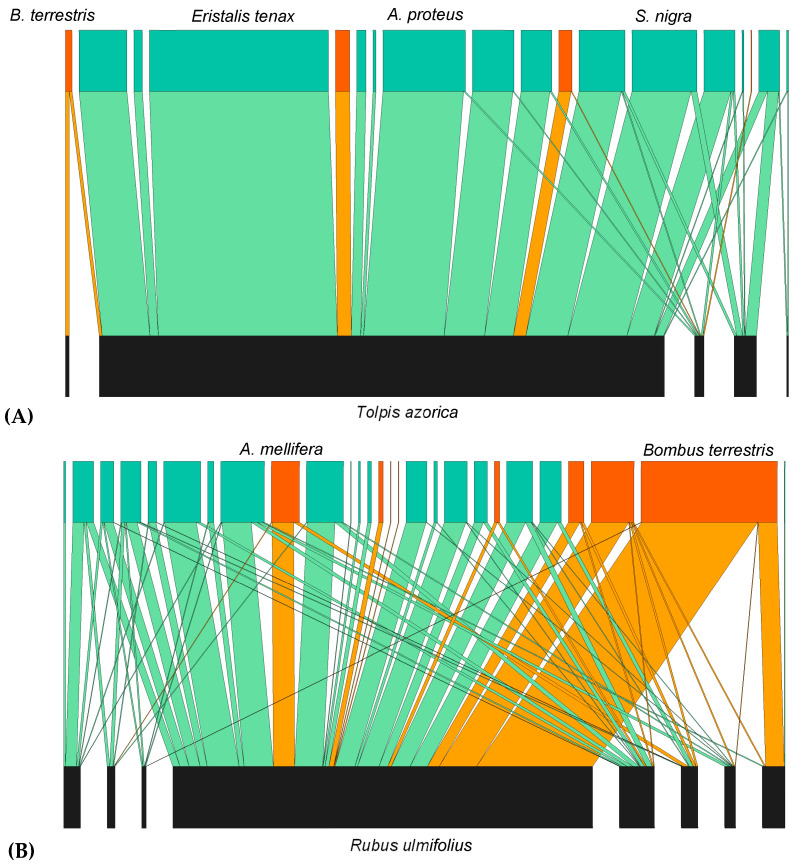
Visitation networks from the preserved (**A**) and the disturbed (**B**) sites at Lomba. Selected plant and pollinator species names are listed at the bottom and at the top, respectively. Pollinators are categorized as native (green) or introduced (orange). Insect visitation to each plant species is proportional to the area of the interaction that connects them.

The visitation networks from preserved and disturbed sites at Lomba and Pico Galhardo showed differences in species richness and composition of plants and insects and in the number and diversity of interactions (Figure 3 and Figure S2, Table 3). Both disturbed sites showed an overall higher plant species richness than the neighboring preserved sites, and in each study area, the average number of links per species was higher in the disturbed site. Interestingly, most visits were directed to one plant species at all study sites. At the preserved sites, the most visited were the endemic plant species *Tolpis azorica* (at Lomba) and *Hypericum foliosum* (at Pico Galhardo), while at both disturbed sites, the introduced *Rubus ulmifolius* received the highest number of visits (Figure 3 and Figure S2).

The values of the connectance of the different pollination networks were relatively similar between study areas and sites, but consistent differences between study sites (preserved vs. disturbed) were found for the other selected network metrics (Table 3). For both study areas, nestedness, interaction strength asymmetry, and network-level specialization (H2) in the disturbed site were lower than in the preserved one, and these differences were larger at Pico Galhardo than at Lomba. However, we need to be cautious with the biological interpretation of these results since network metrics may be strongly influenced by sampling effects and network size [47,52,53] (and references therein). Moreover, the relatively low number of communities targeted in this study may not allow generalizations on the wide-scale effects of disturbance on network structural parameters. At all study sites, we found that irrespective of plant species identity and colonization status, flower abundance was an important driver of insect visitation (all rs ≥ 0.92). Also, despite the occurrence of introduced plants, alien pollinators do not seem to be preferentially associated with introduced plants as in preserved sites they mostly visited native ones (over 92.3% of their visits), particularly those with high flower abundance.

## 4. Discussion

In this study, we assessed the impact of anthropogenic disturbance on the plant and pollinator communities and their ecological interactions in native forest areas of an oceanic island (Terceira, Azores). Despite the proximity of our study sites in each study area, the overall flowering plant species richness was higher at disturbed than preserved sites. This most likely happened because the disturbance facilitated the establishment of introduced species, while several native species still managed to cope with those elevated disturbance levels (Table 1). However, human disturbance still led to a significant reduction in flower availability and a drastic change in plant species composition, with alien species becoming dominant. Surprisingly, these changes did not influence flower visitor richness and visitation frequency, but we witnessed a turnover in the identity of the most important pollinator species and a trend towards an increasing number of visits by introduced pollinators at disturbed sites. Similar findings have been observed in other archipelagos, where the direct and indirect effects of human disturbance led to significant changes in the community of flower visitors [54,55,56]. Human disturbance effects are often mediated by changes in local plant species composition, and their outcomes are complex and species-specific since they differently influence the nutrition, health, and fitness of the various flower visitors [57,58,59].

Since the Azorean islands are relatively young (most with ≤2.1 Ma) and the archipelago is remote (distanced 1368 km from the mainland), it is unsurprising that in the Azorean native forests, most visits to flowers are carried out by a limited set of native pollinators. Also, as a consequence of isolation, the native pollinator fauna shows a taxonomic disharmony: many groups of pollinators that are common in the mainland, like floricolous beetles, butterflies, solitary bees, and wasps, are poorly represented in the archipelago [20,44]. Several flower visitor species have been introduced into the Azores following the exponential increase of commerce and touristic activities [15,20,44] but most have not yet been detected or are still uncommon in the native forests. Two important exceptions are the honeybee (*Apis mellifera*) and the buff-tailed bumblebee (*Bombus terrestris*). These introduced species are common in human-managed habitats (pastures and agricultural fields) that surround the native forest fragments, have high dispersal capabilities and wide feeding spectra, and were often observed visiting flowers at the disturbed sites. Both species benefit from human disturbance of native habitats and, due to their ecological dominance, can potentially drive compositional changes in plant and pollinator communities [28,29]. For instance, in the Canary Islands, the honeybee was found to be responsible for reductions in the number of pollinator species via exploitative competition and the disruption of their interactions with native plants, which in turn influenced pollination services and plant reproductive success [25,60,61,62]. In Terceira, there are no records of feral honeybee colonies, but the number of apiaries and hives grew consistently between 2009 and 2018 (increasing from 75 apiaries and 912 hives to 152 apiaries and 1489 hives) [63]. This growth has continued beyond 2018 following the goals outlined in the strategic plan for beekeeping in the Azores, but its ecological consequences remain unknown. Thus, to ascertain the impact of these introduced species on Azorean native biodiversity, additional studies combining observations and experiments are needed.

Interestingly, irrespective of being native or introduced, the plant species with a higher number of flowers in each study site received the highest number of visits by insects. Flower availability thus seems to be a key factor driving pollinator visitation rates, most probably due to the low pollen and nectar resources found in the Azorean native forests. At the disturbed sites, the invasive elm leaf blackberry (*Rubus ulmifolius*) received the most visits from both native and introduced flower visitors. In its native range, this plant has a generalist pollination system, receiving visits from many species from a wide diversity of insect groups [64]. Alien plants with a generalist pollination strategy may easily invade native plant–pollinator networks by benefiting from biotic facilitation mediated through novel interactions with local pollinators. Several alien plant traits, like flower morphology, size and color, pollen and nectar quantity and quality, or the flowers’ accessibility and availability, seem to have a key role in predicting establishment success and the associations with pollinator groups [65,66].

Many island plant–pollinator networks are characterized by a smaller size and having more generalist interactions as a result of low species numbers and the specific ecological and evolutionary processes operating in isolated biotas [67,68]. The small network size and the dominance of generalist interactions were also the case in our study, where even the few Azorean native forest remnants can themselves be considered ecological islands in a remote archipelago. Several theoretical and empirical studies (reviewed in [49]) showed that more connected and nested networks enhance community stability, while nestedness can also have a positive effect on the resilience of mutualistic communities. From this perspective, the observed reduction in nestedness values between preserved and disturbed sites in our study may indicate an increased vulnerability of plant–pollinator interactions to disturbances, leading to diminished community stability in disturbed sites. However, some studies in island ecosystems reported increases in nestedness following alien species introductions, highlighting their role in enhancing community stability aside from the challenges this situation poses to ecological restoration efforts [69,70].

Our findings highlight the important role of several endemic plant species (also coined super-generalists [17]), such as *Hypericum foliosum* and *Tolpis azorica*, which showed very high generalization levels in Azorean pollination networks. These endemic plants are visited by most pollinator species and seem to play a key role as pollen and nectar sources for pollinator populations. Similarly, high generalism was found in the neighboring archipelagos of Madeira and the Canaries, where several endemic plant species are key resource providers for pollinators [62,71,72]. Several endemic pollinator species, such as the hoverflies *Sphaerophoria nigra* and *Xanthandrus azorensis*, also showed high generalization levels, visiting both native and exotic plants. Olesen and colleagues observed that endemic super-generalist plant and pollinator species are common on oceanic islands, attributing this fact to the reduced interspecific competition and the resulting ecological release [17,18]. Island super-generalists contribute significantly to ecosystem stability by enhancing disturbance resistance through their multiple interactions with diverse partners, including rare endemic species. This high redundancy in plant–pollinator interactions strengthens ecosystem resilience, as populations of interacting species can recover more easily after disturbances by switching partners according to availability. Nevertheless, several authors caution that ecosystems relying on a few key generalist species (as often occurs in small islands and islets) face heightened risk since declines or extinctions of these central species could trigger cascading effects with severe consequences for local biodiversity and ecological processes [23]. Recent studies have shown that super-generalist pollinators can also facilitate the establishment and spread of exotic plant species, potentially increasing the vulnerability of island ecosystems to biological invasions. One notable example is the widespread and locally abundant bee *Homalictus fijiensis*, endemic to the Fijian archipelago, which utilizes a broad range of floral hosts, including numerous alien plants [23,73,74,75]. Among these alien plants are the solanoid species *Dissotis rotundifolia*, *Melastoma denticulatum*, and *Solanum torvum*, which are buzz-pollinated in their native ranges but rely exclusively on *H. fijiensis* for pollination through pollen larceny in the Fiji Islands [23,73,74,75]. The facilitated spread of exotic species by native super-generalists represents a critical ecological issue, posing complex conservation challenges that must be addressed through a multidisciplinary framework to effectively halt biodiversity loss on islands.

This study aims to advance our understanding of the effects of disturbance on pollinator diversity and plant–pollinator interactions in oceanic islands, but we acknowledge the limitations of this research. It would be valuable to extend this sampling to the remaining native forest fragments in Terceira and to other Azorean islands, ensuring a broader spatial coverage and a comprehensive analysis of local findings. Also, it would be key to assess the changes in pollinator abundance, diversity, and visitation patterns by different pollinator species across the flowering season.

## 5. Conclusions

Studying plant–pollinator interactions in oceanic islands is fundamental to better understand the drivers of species loss and ecological changes that are affecting these unique ecosystems. In the Azores, the biota of the remaining native forest fragments is facing an extinction debt as a result of habitat destruction, fragmentation, and disturbance [4], as well as from the arrival and spread of exotic species [12]. Our findings revealed substantial differences in species composition, flower abundance, and plant–pollinator interactions between preserved and disturbed sites and showed that alien species integrate into local plant–pollinator networks, benefiting from associations with super-generalists and altering the interaction frequency between native mutualists. This study stresses the need for further research on the potential detrimental effects of alien species at multiple levels of the pollination process aiming to advance our knowledge of the consequences of how they affect the long-term stability and functionality of native forest ecosystems.

It is essential to establish a long-term monitoring program on plant–pollinator interactions across the Azores, encompassing a variety of habitats and invasion intensities. Such an initiative would enhance understanding of the impacts of alien species on native biodiversity and provide guidance for conservation strategies targeting the archipelago’s endemic plant and pollinator taxa.

## Figures and Tables

**Table 1:** Overall and average flowering plant and insect visitor richness from preserved and disturbed sites in the two study areas (Lomba and Pico Galhardo). The average flower availability at each site is also presented. Data shown as mean ± SD.

	LOMBA		PICO GALHARDO	
	Preserved	Disturbed	Preserved	Disturbed
Flowering plant species richness	7	12	9	14
Average flowering plant species richness	2.8 ± 0.9	2.8 ± 1.1	3.5 ± 1.5	3.4 ± 1.3
Average flower availability	51.1 ± 27.5	33.8 ± 12.8	90.8 ± 41.3	55.6 ± 38.4
Insect visitor richness	30	37	25	22
Average insect visitor richness	9.9 ± 2.7	11.0 ± 4.3	4.4 ± 1.9	3.5 ± 2.6

**Table 2:** Flower visitation by different insect groups in preserved and disturbed sites at the two study areas (Lomba and Pico Galhardo). Data are the percentage of visits by each insect group within each site. The percentage of visits made by introduced insects is also indicated.

	LOMBA		PICO GALHARDO	
POLLINATOR GROUPS	Preserved	Disturbed	Preserved	Disturbed
Beetles (Coleoptera)	9.1	6.9	28.7	2.0
Butterflies and moths (Lepidoptera)	6.0	0.6	2.6	0
Hoverflies (Diptera, Syrphidae)	39.7	25.7	15.1	25.0
Other flies (Diptera)	44.2	39.6	51.5	46.4
Ants and wasps (Hymenoptera)	0	3.1	1.1	14.3
Bees and bumblebees (Hymenoptera, Anthophila)	0.9	24.1	1.1	12.2
Introduced insects	5.1	33.5	5.7	15.2

**Table 3:** Pollination network metrics from preserved and disturbed sites in the two study areas (Lomba and Pico Galhardo).

	LOMBA		PICO GALHARDO	
NETWORK METRICS	Preserved	Disturbed	Preserved	Disturbed
Links per species	1.514	2.128	0.968	1.333
Connectance	0.353	0.270	0.200	0.227
Nestedness	18.130	13.584	22.098	14.312
Interaction strength asymmetry	0.404	0.229	0.720	0.207
Specialization (H2)	0.389	0.320	0.507	0.315

## Data Availability

All relevant data are within this paper and in the Supplementary Materials.

## Supplementary Materials

The following are available online at https://www.mdpi.com/article/10.3390/insects16010014/s1, Figure S1: Spatial distribution of study areas and sampling sites, Figure S2: Visitation networks from the preserved and the disturbed sites at Pico Galhardo, Table S1: List of flowering plants recorded in each study site, Table S2: List of the insect visitors recorded in each study site.
