# Supplementary material for: The Effects of Disturbance on Plant–Pollinator Interactions in the Native Forests of an Oceanic Island (Terceira, Azores)
Source: Insects. 2024 Dec 27;16(1):14. doi: 10.3390/insects16010014 (PMC11765644; doi:10.3390/insects16010014)
Supplement: Supplementary file 1 [file insects-16-00014-s001.zip › FIGURE S1.pdf]

**FIGURE S1.** Spatial distribution of the study areas and sampling sites.

Fieldwork was carried out in two study areas, Lomba and Pico Galhardo, which are considered important for biodiversity conservation in the Azores and both include Terceira Natural Park. Lomba (L) is located in Serra de Santa Bárbara, a large native forest fragment (1347 ha), including the highest mountain top of the island (1021 m) and hosting many endemic plants and animals [34–36]. Pico Galhardo (PG) is a small native forest fragment (180 ha) located in the center of the island, which attains the maximum altitude of (726 m), and is also a key area for nature conservation in the Azores [34].

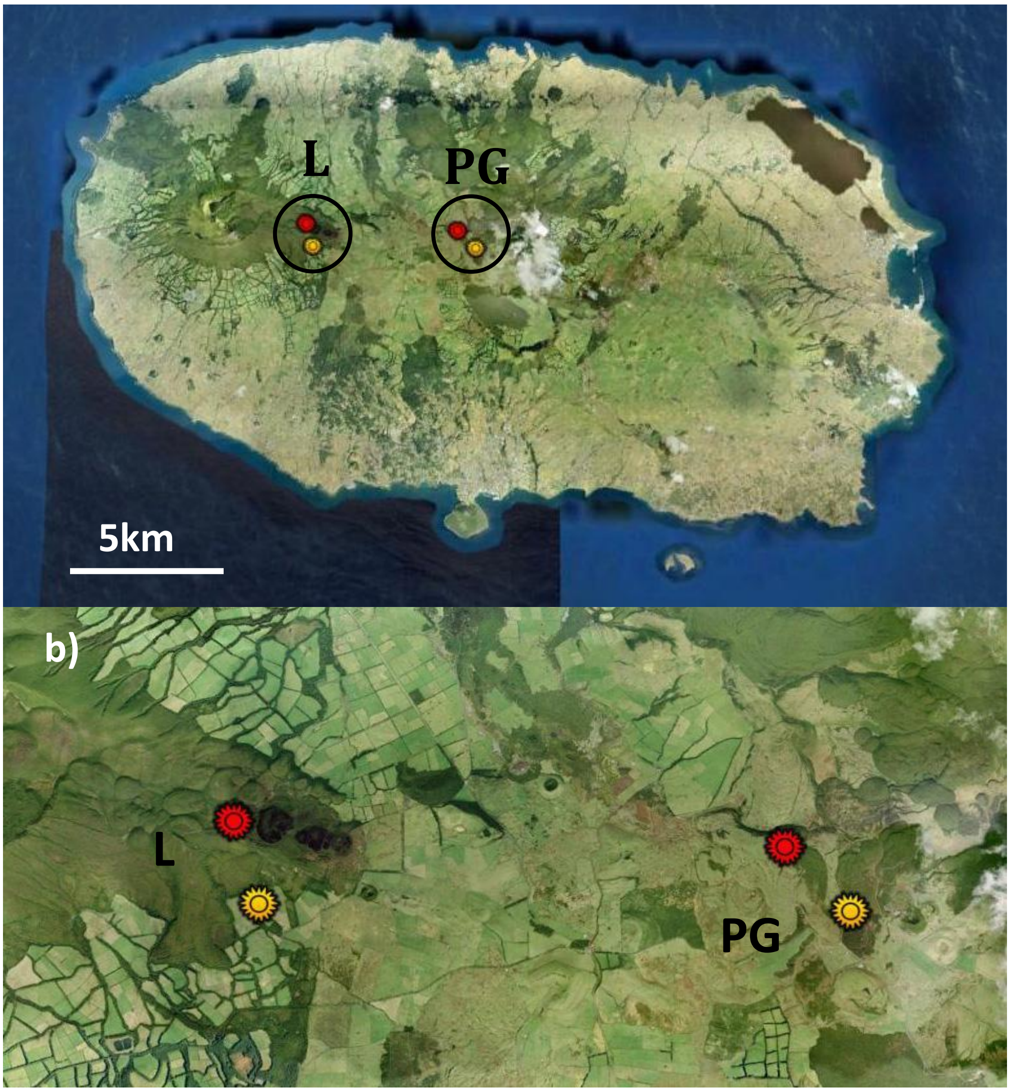


**Figure S1.** Location of the study areas (a) and sampling sites (b) on Terceira island, Azores. The study areas are Lomba (L) and Pico Galhardo (PG). Sampling sites are represented by coloured symbols (red and yellow markings are preserved and disturbed sites, respectively).

We adopted a paired-sampling design by selecting two sampling sites (one well-preserved and one disturbed) in each study area. These sampling sites were set close to each other (~500m) to minimize geographic distance effects on community composition, but they differed between each other in plant structure and composition due to a higher number of introduced plant species in the disturbed site.

The locations of the study sites are as follows (see also Figure S1):

- Lomba, preserved site: UTM 26S 04752E 42874N
- Lomba, disturbed site: UTM 26S 04752E 42866N
- Pico Galhardo, preserved site: UTM 26S 04803E 42871N
- Pico Galhardo, disturbed site: UTM 26S 04806E 42867N
