# Supplementary material for: The Effects of Disturbance on Plant–Pollinator Interactions in the Native Forests of an Oceanic Island (Terceira, Azores)
Source: Insects. 2024 Dec 27;16(1):14. doi: 10.3390/insects16010014 (PMC11765644; doi:10.3390/insects16010014)
Supplement: Supplementary file 1 [file insects-16-00014-s001.zip › FIGURE S2.pdf]

FIGURE S2. Visitation networks from the preserved and the disturbed sites at Pico Galhardo.

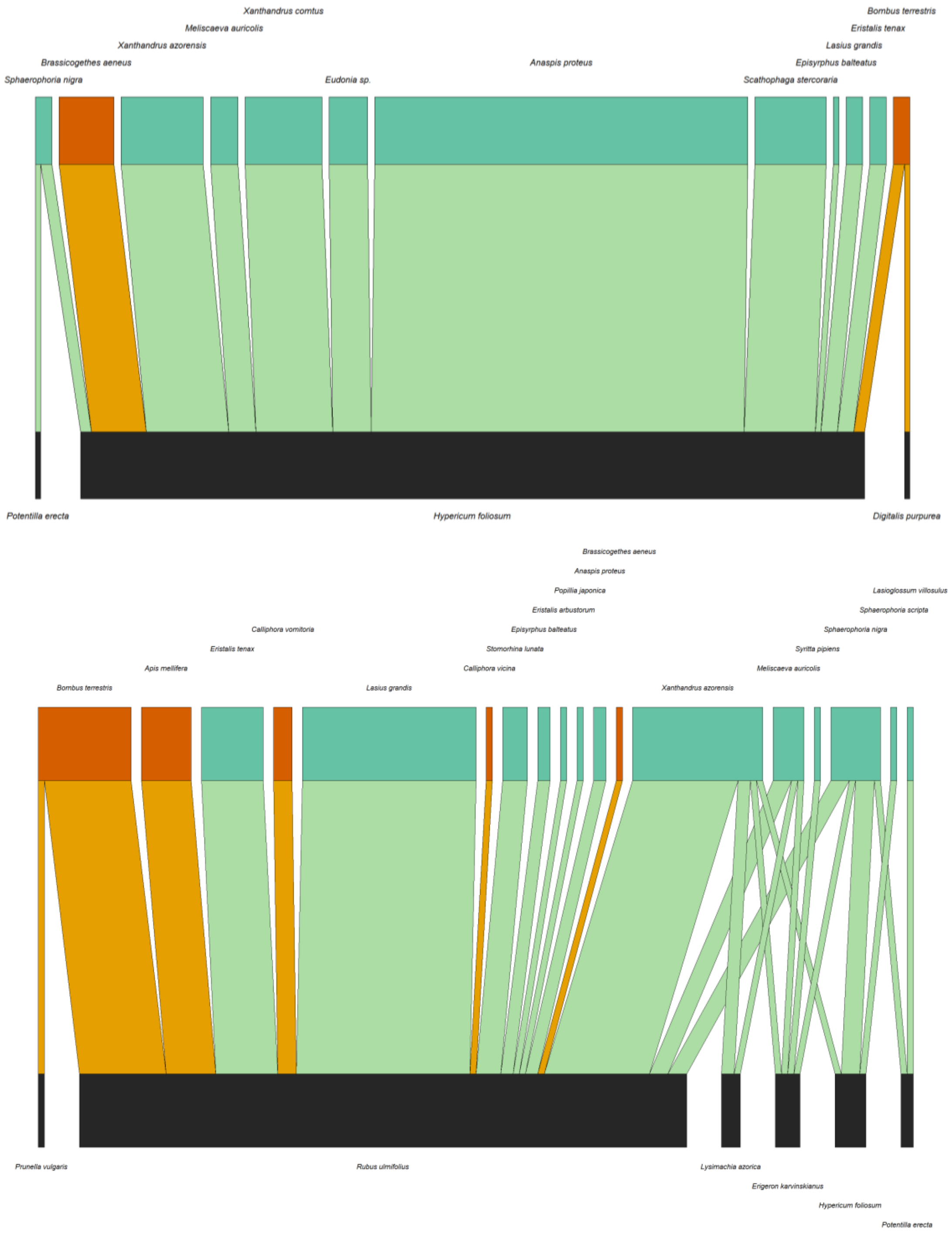

Figure S2. Visitation networks from the preserved (A) and the disturbed (B) sites at Pico Galhardo. Plant species are listed at the bottom, while pollinator species are positioned at the top. Pollinators are categorized as native (green) or introduced (orange). Insect visitation to each plant species is proportional to the area of the interaction that connects them.
