# Supplementary material for: The Effects of Disturbance on Plant–Pollinator Interactions in the Native Forests of an Oceanic Island (Terceira, Azores)
Source: Insects. 2024 Dec 27;16(1):14. doi: 10.3390/insects16010014 (PMC11765644; doi:10.3390/insects16010014)
Supplement: Supplementary file 1 [file insects-16-00014-s001.zip › TABLE S1.pdf]

Table S1. List of flowering plants recorded in each study site. The distribution status of each species [endemic (END), non-endemic native (NAT), introduced (INT), naturalized or doubtful] follows the most recent checklist on Azorean biodiversity [20].

Plant Species	Distribution status	Lomba		Pico Galhardo	
		Preserved	Disturbed	Preserved	Disturbed
Calluna vulgaris (L.) Hull	NAT	x			
Crepis capillaris (L.) Wallr.	INT			x	x
Digitalis purpurea L.	INT			x	x
Erigeron karvinskianus D.C.	INT				x
Hydrangea macrophylla (Thunb.) Ser.	INT		x		
Hypericum foliosum Aiton	END		x	x	x
Hypericum humifusum L.	NAT				x
Hypochaeris radicata L.	INT	x	x		
Lobelia urens L.	INT			x	x
Lotus pedunculatus Cav.	INT	x		x	x
Lysimachia azorica Hornem. ex Hook.	END		x	x	x
Lythrum junceum Banks & Sol.	INT				x
Persicaria capitata (Buch.-Ham. ex D.Don) H.Gross	INT		x		x
Potentilla erecta (L.) Räusch.	NAT	x	x	x	x
Prunella vulgaris L.	Doubtful	x	x	x	x
Ranunculus repens L.	Naturalized		x		
Rubus ulmifolius Schott	INT		x		x
Scrophularia scorodonia L.	INT		x		
Thrincia saxatilis (Lam.) Holub & Moravec	Doubtful		x	x	
Tolpis azorica (Nutt.) P. Silva	END	x			
Trifolium repens L.	INT		x		
Vaccinium cylindraceum Sm.	END	x			
