# Supplementary material for: The Effects of Disturbance on Plant–Pollinator Interactions in the Native Forests of an Oceanic Island (Terceira, Azores)
Source: Insects. 2024 Dec 27;16(1):14. doi: 10.3390/insects16010014 (PMC11765644; doi:10.3390/insects16010014)
Supplement: Supplementary file 1 [file insects-16-00014-s001.zip › TABLE S2.pdf]

Table S2. List of the insect visitors recorded in each study site, with indication of their taxonomic group and distribution status. The distribution status of each species [endemic (END), non-endemic native (NAT) or introduced (INT)] follows the most recent checklist on Azorean terrestrial arthropod biodiversity [43].

Order	Family	Species	Distribution status	Lomba		Pico Galhardo	
				Preserved	Disturbed	Preserved	Disturbed
Coleoptera	Carabidae	*Gen sp1*	-	x			
		Gen sp2	-	x			
	Nitidulidae	*Brassicogethes aeneus* (Fabricius, 1775)	INT			x	x
	Scarabaeidae	*Popillia japonica* Newman, 1838	INT		x		x
	Scraptiidae	*Anaspis proteus* Wollaston, 1854	NAT	x	x	x	x
Diptera	Agromyzidae	*Gen sp1*	-			x	
	Anthomyiidae	*Gen sp1*	-	x	x	x	
	Chloropidae	*Gen sp1*	-			x	
	Calliphoridae	*Calliphora vicina* Robineau-Desvoidy, 1830	INT	x	x		x
		Calliphora vomitoria (Linnaeus, 1758)	INT		x		x
		Lucilia sericata (Meigen, 1826)	INT	x	x		
		Pollenia rudis (Fabricius, 1794)	INT	x	x		
		Stomorhina lunata (Fabricius, 1805)	NAT	x	x		x
	Dolichopodidae	*Gen sp1*	-	x	x	x	x
	Empididae	*Gen sp1*	-	x			
	Ephydridae	*Gen sp1*	-			x	
	Muscidae	*Eudasyphora cyanella* (Meigen, 1826)	INT	x	x	x	
		Gen sp1	-	x	x	x	x
		Hydrotaea dentipes (Fabricius, 1805)	INT		x	x	
		Musca osiris Wiedemann, 1830	INT	x	x		
		Stomoxys calcitrans (Linnaeus, 1758)	INT	x			
	Phoridae	*Gen sp1*	-			x	x
	Sepsidae	*Sepsis biflexuosa* Strobl, 1893	NAT	x	x	x	
		Sepsis fulgens Meigen, 1826	NAT?	x	x	x	x
		Sepsis thoracica (Robineau-Desvoidy, 1830)	NAT	x	x	x	x
	Scathophagidae	*Scathophaga stercoraria* (Linnaeus, 1758)	NAT	x	x	x	
	Sciaridae	*Gen sp1*	-		x		

	Syrphidae	*Episyrphus balteatus* (De Geer, 1776)	NAT	x	x	x	x
		Eristalis arbustorum (Linnaeus, 1758)	NAT	x	x		x
		Eristalis tenax (Linnaeus, 1758)	NAT	x	x	x	x
		Eupeodes corollae (Fabricius, 1794)	NAT	x	x		
		Meliscaeva auricolis (Meigen, 1822)	NAT		x	x	x
		Myathropa florea (Linnaeus, 1758)	NAT		x		
		Sphaerophoria nigra Frey, 1945	END	x	x	x	x
		Sphaerophoria scripta (Linnaeus, 1758)	NAT	x	x		x
		Syritta pipiens (Linnaeus, 1758)	NAT	x	x		x
		Xanthandrus azorensis Frey, 1945	END	x	x	x	x
		Xanthandrus comtus (Harris, 1780)	NAT		x	x	
	Tephritidae	*Gen sp1*	-	x	x	x	
Hymenoptera	Apidae	*Apis mellifera* Linnaeus, 1758	INT		x		x
		Bombus terrestris (Linnaeus, 1758)	INT	x	x	x	x
		Lasioglossum villosulum (Kirby, 1802)	NAT	x	x		x
		Megachile cetuncularis (Linnaeus, 1758)	NAT		x		
	Formicidae	*Lasius grandis* Forel, 1909	NAT		x	x	x
	Vespidae	*Vespula germanica* (Fabricius, 1793)	NAT		x		
Lepidoptera	Crambidae	*Eudonia sp.*	END	x	x	x	
	Noctuidae	*Gen sp1*	-		x		
	Nymphalidae	*Hipparchia azorina* (Strecker, 1899)	END	x			
	Pieridae	*Pieris brassicae azorensis* Rebel, 1917	END		x		
